# [Retracted] 8-bromo-7-methoxychrysin induces apoptosis by regulating Akt/FOXO3a pathway in cisplatin-sensitive and resistant ovarian cancer cells

**DOI:** 10.3892/mmr.2026.13796

**Published:** 2026-01-12

**Authors:** Qing Ding, Yi Chen, Qing Zhang, Yanling Guo, Zhi Huang, Liqing Dai, Sudan Cao

Mol Med Rep 12: 5100–5108, 2015; DOI: 10.3892/mmr.2015.4039

Following the publication of the above paper, it was drawn to the Editor's attention by a concerned reader that certain of the western blot data shown in Fig. 2C on p. 5104 were strikingly similar to data appearing in different form in another article written by different authors at different research institutes that had already been published in the journal *Oncogene.* An independent analysis of the data in this paper made by the Editorial Office further revealed that other western blot data in the same figure had appeared in a number of other papers written by different authors that had also been published previously; furthermore, there were internally duplicated data among the western blots in Fig. 1, Fig. 2, Fig. 3, Fig. 4, Fig. 5, and potential anomalies concerning the assembly of the data in these figures.

Owing to the fact that the contentious data mentioned above had already apparently been published previously, the Editor of *Molecular Medicine Reports* has decided that this paper should be retracted from the Journal. The authors were asked for an explanation to account for these concerns, but the Editorial Office did not receive a reply. The Editor apologizes to the readership for any inconvenience caused.

